# Fluorescence in situ hybridization investigation of potentially pathogenic bacteria involved in neonatal porcine diarrhea

**DOI:** 10.1186/1746-6148-10-68

**Published:** 2014-03-14

**Authors:** Beata Jonach, Mette Boye, Anders Stockmarr, Tim Kåre Jensen

**Affiliations:** 1 National Veterinary Institute, Technical University of Denmark, Bülowsvej 27, Frederiksberg 1870, Denmark; 2 Department of Applied Mathematics and Computer Science, Technical University of Denmark, Matematiktorvet, Building 303B Kgs, Lyngby, Denmark

**Keywords:** *E. coli*, *Enterococcus*, Fluorescence in situ hybridization, Neonatal diarrhea, Potentially pathogenic bacteria

## Abstract

**Background:**

Neonatal diarrhea is a multifactorial condition commonly present on pig farms and leads to economic losses due to increased morbidity and mortality of piglets. Immature immune system and lack of fully established microbiota at birth predispose neonatal piglets to infection with enteric pathogens. The microorganisms that for decades have been associated with enteritis and diarrhea in suckling piglets are: rotavirus A, coronavirus, enterotoxigenic *Escherichia coli* (ETEC), *Clostridium perfringens* type C, *Cryptosporidium* spp*.*, *Giardia* spp., *Cystoisospora suis* and *Strongyloides ransomi.* However, in recent years, the pig industry has experienced an increased number of neonatal diarrhea cases in which the above mentioned pathogens are no longer detected. Potentially pathogenic bacteria have recently received focus in the research on the possible etiology of neonatal diarrhea not caused by common pathogens. The primary aim of this study was to investigate the role of *E. coli*, *Enterococcus* spp., *C. perfringens* and *C. difficile* in the pathogenesis of neonatal porcine diarrhea with no established casual agents. Fluorescence in situ hybridization with oligonucleotide probes was applied on the fixed intestinal tissue samples from 51 diarrheic and 50 non-diarrheic piglets collected from four Danish farms during outbreaks of neonatal diarrhea not caused by well-known enteric pathogens. Furthermore, an association between the presence of these bacteria and histological lesions was evaluated.

**Results:**

The prevalence of fluorescence signals specific for *E. coli*, *C. perfringens* and *C. difficile* was similar in both groups of piglets. However, *Enterococcus* spp. was primarily detected in the diarrheic piglets. Furthermore, adherent bacteria were detected in 37 % diarrheic and 14 % non-diarrheic piglets. These bacteria were identified as *E. coli* and *Enterococcus* spp. and their presence in the intestinal mucosa was associated with histopathological changes.

**Conclusions:**

The results of this study showed that simultaneous colonization of the intestinal mucosa by adherent non-ETEC *E. coli* and *Enterococcus* spp. can be involved in the pathogenesis of neonatal porcine diarrhea. These bacteria should be considered in diagnosis of diarrhea in piglets, when detection of common, well-known enteric agents is unsuccessful.

## Background

Neonatal diarrhea is a major problem that leads to reduced weight gain, increased morbidity and mortality among piglets and has, in consequence, an economic impact on the swine industry. Diarrhea in suckling piglets is often of infectious nature and results from interactions between pathogens, an immature immune system and the environment. In the past, a number of microorganisms have been associated with pig neonatal enteritis i.e. rotavirus A, coronavirus, enterotoxigenic *Escherichia coli* (ETEC), *Clostridium perfringens* type C, *Cryptosporidium* spp., *Giardia* spp., *Cystoisospora suis* and *Strongyloides ransomi*[1,2]. However, in recent years, the pig industry in many countries has experienced an increased number of neonatal diarrhea cases not caused by the above mentioned pathogens [3-5]. Considering contribution of bacterial agents, identification of the etiology of this diarrhea becomes challenging and problematic. The gastrointestinal tract hosts diverse population of microorganisms composed of commensals and potentially pathogenic bacteria. These bacteria interact constantly in a synergistic and competitive manner and any disturbances in this microbial community can cause bacterial overgrowth and disease [6]. Due to immunodeficiency and incomplete gut microbiota at birth, the neonatal piglets are particularly vulnerable to infectious enteric diseases [7]. However, recognition of the pathogenic bacteria in gut population and distinguishing from non-harmful microbiota is difficult and restricted by the fact that bacterial diversity is not yet fully explored and the pathogenic bacteria are also present in clinically healthy animals. Nevertheless, a few potentially pathogenic bacteria that are considered to be part of the normal intestinal microbiota, have been recently studied as a potential cause of neonatal enteritis. The aim of this study was to investigate the role of the following bacteria: *E. coli*, *Enterococcus* spp., *C. perfringens* and *C. difficile* in naturally occurring pig neonatal diarrhea not caused by known enteric pathogens. For detection of these bacteria we used fluorescence in situ hybridization (FISH) targeting ribosomal RNA as this is a rapid and reliable method that allows a direct visualization, identification and spatial localization of bacteria within fixed tissue samples [8,9].

## Materials and methods

### Animals

In total 51 diarrheic and 50 non-diarrheic piglets aged 3–7 days were included in this study. The piglets were selected from four commercial Danish swine herds presenting high standards of management and housing. Diarrhea of unknown etiology (not caused by either enterotoxigenic *E. coli*, *C. perfringens* type C, rotavirus A, coronavirus or parasites) and poorly responding to antibiotic therapy was present in at least 30 % litters in each herd for a period of minimum six months. From each herd 11–14 diarrheic and 12–13 non-diarrheic piglets (age matched) from several litters (maximum two piglets per litter) were selected. The diarrheic piglets had diarrhea for at least 2 days prior to euthanasia and were selected from the litters with the highest prevalence of diarrhea. The non-diarrheic piglets did not have diarrhea at any time and were selected from the litters with no diarrhea or very low prevalence of diarrhea. For further details on the selection of herds and piglets the reader is referred to Kongsted *et al*.; 2013 [3].

### Intestinal samples and histopathology

The samples of duodenum, jejunum, ileum and spiral colon were collected during necropsies, fixed in 10 % neutral buffered formalin for at least 48 h, embedded in paraffin wax and cut at 3 μm. From each piglet one section of duodenum, two sections of jejunum, two sections of ileum and two sections of colon were examined histopathologically and by FISH. Sections for histopathology were stained with hematoxylin and eosin (HE) according to standard laboratory procedures and investigated microscopically for the presence of histological lesions, which is described in Kongsted *et al*. [3]. Sections for FISH were mounted on SuperFrost®White slides (Mensel-Gläser, Braunschweig, Germany). Prior to hybridization the sections were deparaffinized in xylene (2 × 2 min), treated with 99.9 % ethanol (2 × 2 min) and air dried.

### Fluorescence in situ hybridization (FISH)

FISH was carried out with oligonucleotide probes targeting bacterial 16S or 23S rRNA as listed in Table 1. For each animal FISH was performed as double hybridization with two probes: a general bacterial probe targeting *Domain bacteria* (labeled with fluorescein isothiocyanate) and one of the specific probes directed against *E. coli, Enterococcus* spp.*, C. perfringens* or *C. difficile* (labeled with the cyanine dye Cy3).

**Table 1:** List of the oligonucleotide probes used in this study*

**Target bacteria**	**Name of the probe**	**Target sequence (5’-3’)**	**Target region of rRNA**	**References**
*Domain bacteria*	S-D-Eub-0338	5’-GCTGCCTCCCGTAGGAGT-3’	16S	[10]
*Escherichia coli*	S-S-E.coli-1161	5’-GCATAAGCGTCGCTGCCG-3’	23S	[11]
*Enterococcus spp.*	S-G-Enteroco-184	5’-CAAATCAAAACCATGCGG-3’	16S	[12]
*Clostridium perfringens*	S-S-C.perfring-1	5’-TGGTTGAATGATGATGCC-3’	16S	[13]
*Clostridium difficile*	S-S-C.diff-193	5’-TGTACTGGCTCACCTTTG-3’	16S	[14]

* The probes were purchased from Eurofins MWG Operon, Ebersberg, Germany.

Hybridization was carried out overnight with each probe in concentration of 5 ng/μl hybridization buffer (1 M Tris- pH 7.2, 5 M NaCl, 10 % sodium dodecyl sulphate) at 45 °C. The sections were then washed 3 × 3 min in pre-warmed (45 °C) hybridization buffer and 3 × 3 min in pre-warmed (45 °C) washing buffer (1 M Tris- pH 7.2, 5 M NaCl). The samples were finally rinsed in water, air dried and mounted in Vectashield (Vector Laboratories, Burlingame, CA, USA) for epifluorescence microscopy.

### Epifluorescence microscopy

Microscopic examination was performed using a Zeiss Axioimager M1 epifluorescence microscope equipped with a 120-W HBO lamp. The following filter sets were applied for detection of fluorescence signals: filter set 38 for detection of fluorescein, filter set 43 for detection of Cy3 and filter set 24 for simultaneous detection of green and red fluorescence. Micrographs were taken using an AxioCam MRm version 3 FireWire monocrome camera and AxioVision software, version 4.5 (Carl Zeiss, Oberkochen, Germany).

### Evaluation of fluorescence signals

**Figure 1:**
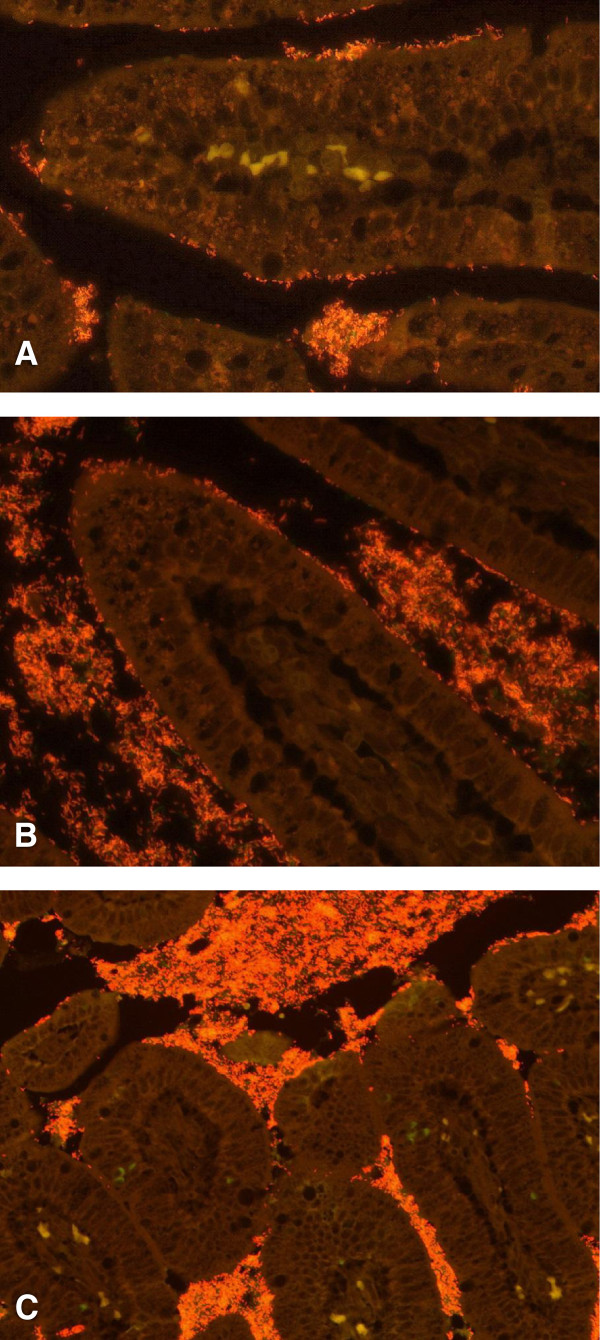
**Representative micrographs showing semi-quantitative scores of the fluorescence signals in the microscopic field. A**. + small amount of bacteria (red), magnification 400x. **B**. ++ moderate amount of bacteria (red), magnification 400x. **C**. +++ large amount of bacteria (red), magnification 400×.

Evaluation of fluorescence signals in all intestinal regions from each piglet was performed blindly by the first author using a 40× objective and included determination of the amount of bacterial cells and their distribution in the tissue section. A positive score for particular bacteria was given when the hybridization signals were clearly specific and distinguishable as bacterial cells and could be identified with the specific probe. The amount of bacteria in each intestinal region was scored in a semi-quantitative manner according to the following scale: + small amount, ++ moderate amount, +++ large amount of bacteria. A score of + was given to the tissue specimens in which the number of bacterial cells present in the microscopic field was in a countable range and/or bacterial cells were clustered in small groups (Figure 1A). When amount of bacterial cells was out of a countable range and the bacterial cells were grouped over larger areas of the microscopic field, the tissue specimen received ++ (Figure 1B). A score of +++ was given to the tissue specimens in which bacteria were present in a strikingly large amount as well as when single bacterial cells could no longer be distinguished due to high concentration of bacteria and the fluorescence signals were fused together (Figure 1C). Evaluation of the total amount of bacteria in the small intestines was based on the highest score given for a particular intestinal region.

### Statistical analyses

Presence and localization of the investigated bacteria data were modeled with a mixed effects model, where the logit of the probability of bacterial presence and location was allowed to depend on diarrheic status, presence of histological lesions and age of the piglets. Herd was included as a random effect. Spearman rank correlation was calculated between the presence of pathogens and histological lesions. Furthermore, the amounts of the bacteria were analyzed with a multinomial effects model where herd, diarrheic status, age and presence of histological lesions were explanatory variables, using neural networks for parameter estimation. Model reduction was performed using the likelihood ratio method.

## Results

**Figure 2:**
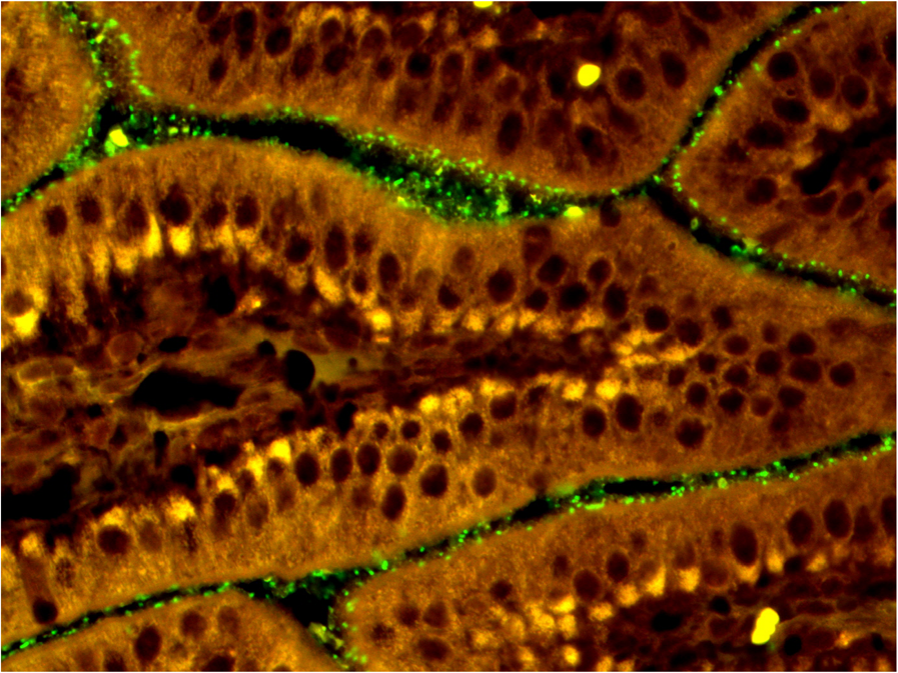
**Section of the jejunum from a diarrheic piglet hybridized with the general bacterial probe.** Mixed colony of cocci and coliform bacteria (green) intimately adhere to the villous epithelial cells (brown). Magnification 400x.

Hybridization with the universal probe targeting *Domain bacteria* showed bacteria of various morphotypes in all piglets. In vast majority of piglets the largest amounts of bacteria were present within the intestinal content in the colon. However, if the content was missed during tissue preparation, the amounts of bacteria seen in the colon were lower compared to other regions of the intestine. In the small intestines larger amounts of bacteria were usually seen in the ileum compared to the jejunum and duodenum. In the latter one the bacterial cells were present only in small amounts in all animals. Bacteria were spread in the intestinal lumen and the space between the villi. Additionally, in 37 % of diarrheic and 14 % of non-diarrheic piglets bacterial cells adhered to the intestinal epithelium lining the villi (Figure 2). There was no specific fluorescence seen in intracellular regions, however, in two diarrheic piglets with diffuse necrotic changes in the mucosa the fluorescence signals for *Domain bacteria* were seen in large amounts within the necrotic tissue.

**Figure 3:**
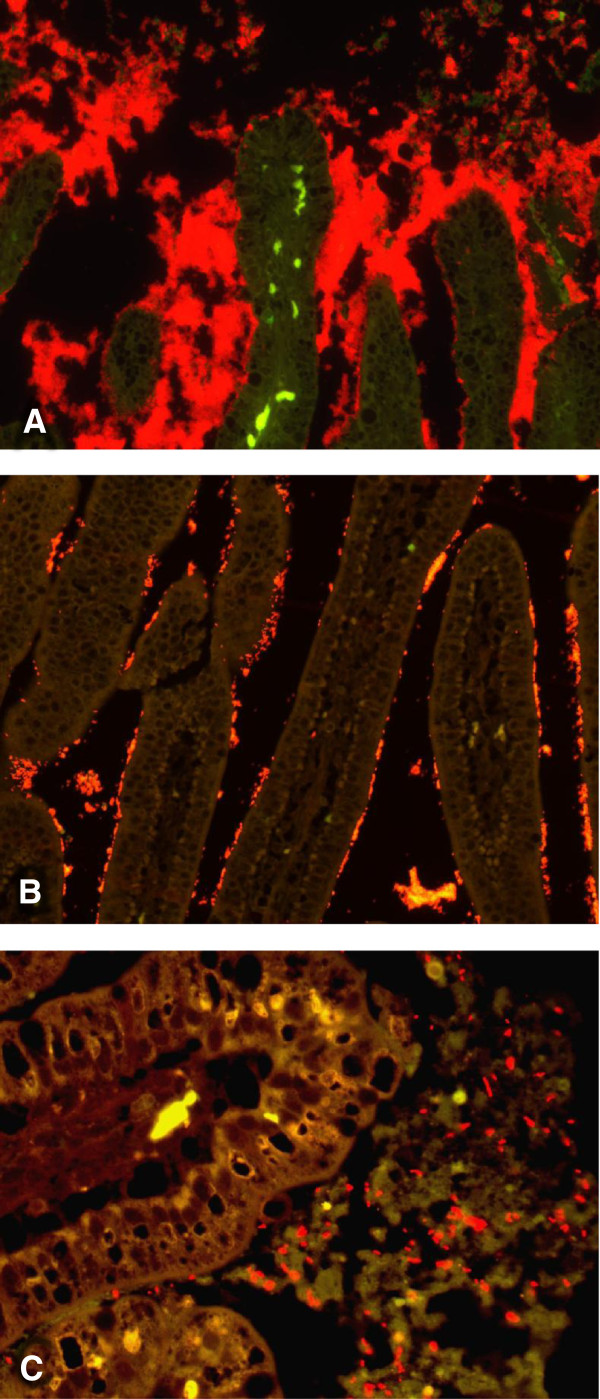
**Intestinal tissue sections colonized by *E. coli. *A**. Jejunum from a diarrheic piglet. Large fluorescence mass consisting of *E. coli* (red) present in the intestinal lumen, between the villi (green) and attached to the villous epithelium. The yellowish-green fluorescence present in the centrally located villous results from autofluorescence of the erythrocytes. Magnification 200x. **B**. Jejunum from a diarrheic piglet. *E. coli* cells (red) adhere to the intestinal epithelium lining the villi (brown). Magnification 200x. **C**. Jejunum of a non-diarrheic piglet. Small amounts of *E. coli* are present in the intestinal lumen with no adhesion to the tissue. Magnification 400x.

In the small intestines positive signals for *E. coli* were seen in 88 % of diarrheic and 80 % of non-diarrheic piglets. *E. coli* were present mostly in the intestinal lumen and luminal space between the villi. The diarrheic piglets had more frequently large amounts of *E. coli* compared to the non-diarrheic piglets (p < 0.05). Additionally, in 33 % of diarrheic and 14 % of non-diarrheic piglets *E. coli* adhered to the villous epithelial cells (Figure 3). The majority of the diarrheic piglets (59 %) with adherent *E. coli* originated from one herd. The presence of *E. coli* was positively correlated with villous atrophy (r_s_: 0.20, p < 0.05) and neutrophil infiltration (r_s_: 0.20, p < 0.05) regardless of the diarrheic status.

In the colon positive fluorescence signals for *E. coli* were present within the intestinal content or in the lumen in all piglets. Additionally, in two diarrheic piglets the bacteria adhered to the luminal surface of the colonic epithelium that showed excessive extrusion and deformation of the enterocytes and in one non-diarrheic piglet *E. coli* adhered to the luminal colonic enterocytes that otherwise appeared normal.

**Figure 4:**
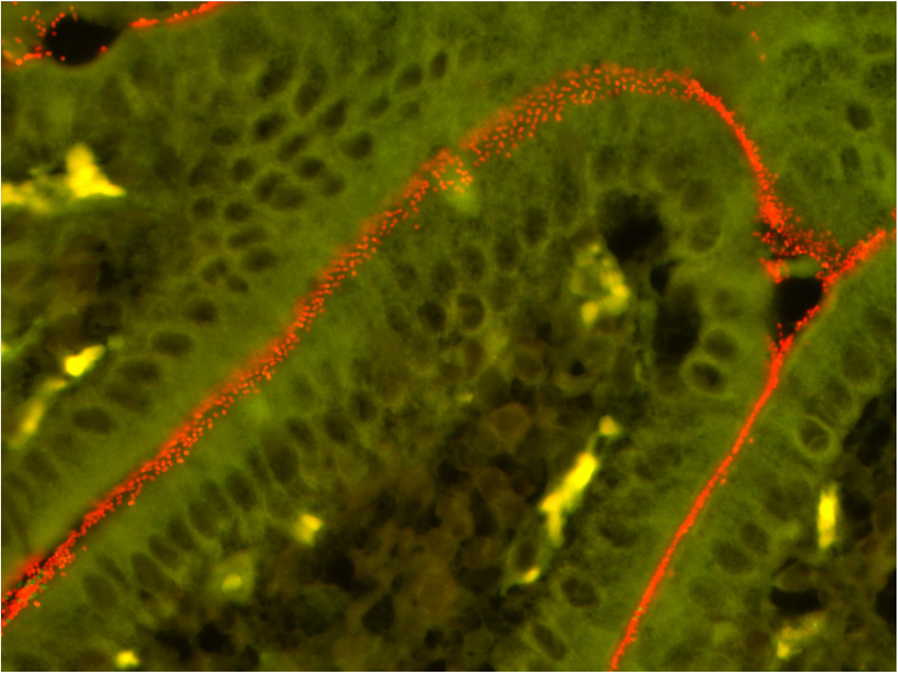
**Section of the jejunum from a diarrheic piglet hybridized with the probe specific for *Enterococcus spp.*** Enterococci (red) adhere to the epithelium lining the villi (greenish). Magnification 400x.

*Enterococcus* spp. cells were detected in the small intestines of 45 % of diarrheic and 8 % of non-diarrheic piglets and their presence was positively correlated with villous atrophy (r_s_: 0.26, p < 0.01). Adherent enterococci were seen in association with the small intestinal epithelium lining the villi in 27 % of diarrheic and 2 % of non-diarrheic piglets (Figure 4). In the majority of cases adherent enterococci were seen in the small intestine of those diarrheic piglets that also had adherent *E. coli* (r_s_: 0.58, p < 0.0001). Presence of adherent enterococci was associated with mild epithelial lesions (r_s_: 0.45, p < 0.05). In the piglets aged 3 days the presence of adherent enterococci was positively correlated with neutrophil granulocyte infiltration, however the correlation was negative in the piglets aged 4–7 days.

Positive fluorescence signals for *C. perfringens* were detected in 73 % of diarrheic and 78 % of non-diarrheic piglets. These bacteria were mostly present in the intestinal lumen, however, in 20 % of diarrheic and 30 % of non-diarrheic animals the bacteria were found within the mucus layer and in direct contact with the intestinal epithelium. Additionally, in the ileum and colon of one diarrheic piglet *C. perfringens* cells were seen in large amounts within the necrotic tissue alongside other bacteria detected by the general bacterial probe. Otherwise there was no significant correlation between the presence and location of *C. perfringens* in the intestinal tissue and histological lesions. *C. difficile* cells were detected in small amounts within the intestinal content of the colon in 65 % of diarrheic and 70 % of non-diarrheic piglets. Single *C. difficile* cells were also present in the lumen of the small intestine in 12 % of diarrheic and 14 % of non-diarrheic piglets. There was no correlation between the presence of *C. difficile* and histological lesions and diarrhea.

**Figure 5:**
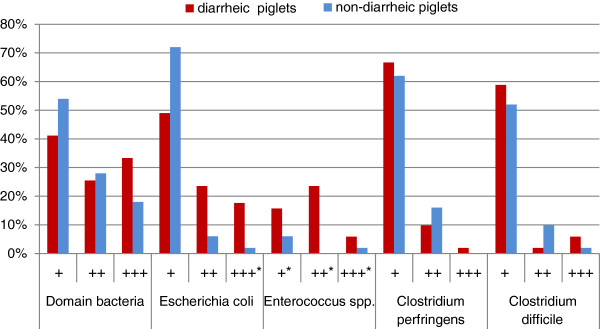
**Semi-quantitative analyses of the fluorescence signals in the intestines of 51 diarrheic and 50 non-diarrheic piglets.** +: Small amounts, ++: moderate amounts, +++: large amounts of bacteria. *depicts significant positive correlation between amount of bacteria and diarrhea (p-value <0.05).

The results of semi-quantitative score are shown in Figure 5.

## Discussion

The objective of this study was to elucidate the role of *E. coli*, *Enterococcus* spp., *C. perfringens* and *C. difficile* in neonatal porcine diarrhea with no previously established etiology. We used FISH method for investigation of the prevalence, abundance and location of these potentially pathogenic bacteria in the intestinal tissue because a direct visualization of microorganisms helps to determine their association with the mucosa surface and may therefore have a potential value in elucidating their role in the disease. Sensitivity of FISH method for detection of bacteria depends on many factors including metabolic activity of microbial cells and availability of target sequences in the tissue samples. It has been shown that in natural samples the fluorescence signal intensity may be too low for identification of microorganisms [15]. However, because the intestinal microbiota are expected to express high metabolic activity and possess high content of rRNA, the FISH method seems to be an appropriate approach for in situ detection of enteric bacteria. In order to better control for eventual false negative results due to methodological problems we decided to apply a general bacterial probe simultaneously with the specific probe on all tissue specimens. As the FISH results of hybridization with the general probe were satisfactory, we consider sensitivity of FISH performed in this investigation to be high.

According to informations provided at probeBase [16], three oligonucleotide probes used in this study: *Enterococcus* spp, *C. perfringens* and *C. difficile* probes, were shown to be highly specific and reliable for detection of these particular bacteria. However, the *E. coli* probe used in this investigation was noted to be unable to target all *E. coli* strains. Additionally, the target sequence of this probe was shown to match other enterobacteria; however these were not relevant as swine pathogens.

Quantification of microbial community detected by FISH in the intestinal tissue samples is not possible when the bacteria are present in high concentration as it hinders distinction of fluorescence signals from single bacterial cell. Therefore, evaluation of the amount of bacteria was done in a semi-quantitative manner, based on the subjective judgment of the investigator.

Adhesion of bacteria to the epithelial cells is believed to be an initial and pivotal event in the pathogenesis of most bacterial enteric infections and is necessary for allowing bacteria to survive and persist in a continuously moving environment and to defeat host defense mechanisms [6]. In this study adherent bacteria were seen in 37 % of diarrheic and 14 % of non-diarrheic piglets. The non-diarrheic piglets were collected from the same herds as the diarrheic ones. Therefore, it cannot be excluded that some of the non-diarrheic piglets were in the initial phase of infection, thus expressed similar composition of eventual pathogens as the diarrheic piglets. In situ hybridization with the specific probes identified these bacteria as *E. coli* and/or *Enterococcus* spp. and there was a significant positive correlation between adherence of these bacteria and diarrhea. Furthermore, large amounts of *E. coli* were seen in significantly higher number of diarrheic piglets compared to non-diarrheic animals. Overgrowth and colonization of the mucosal surface by *E. coli* suggest its involvement in diarrhea. The piglets involved in this study were negative for enterotoxigenic *E. coli* (ETEC) [3], which are considered to be the most common cause of pig neonatal diarrhea and for which the ability to attach to and colonize the intestinal epithelium is believed to be a hallmark virulence trait [17]. However, *E. coli* strains other than ETEC have also been shown to be able to adhere to the intestinal mucosa surface and cause diarrhea [18]. In addition, attaching and effacing *E. coli* (AEEC) that have ability to cause attaching and effacing lesions in the gut mucosa, have been associated with diarrhea in domestic animals including pigs [19,20]. Therefore, further work will be done towards identifying and defining the pathogenicity of adherent *E. coli* found in this study.

We observed a significant positive correlation between the presence of *E. coli* and histomorphological changes in the intestinal mucosa. Villous atrophy is a common condition in diarrheal diseases and in suckling piglets this is primarily associated with viral or parasitic infections [21]. However, some reports have shown that shortening of villi and epithelial lesions can follow colonization of the mucosa by *E. coli*[22,23]. Since the piglets included in this study were thought to be free from infection with commonly known pathogens, at this stage of investigation it is difficult to conclude whether overgrowth and colonization of the mucosa by *E. coli* was a primary event in the pathogenesis of villous atrophy or was secondary to infection with other, yet unidentified microorganisms, which cause alteration in the intestinal villi. Further studies are currently being conducted in order to determine the etiology of the presently described diarrhea.

Enterococci are commensal bacteria in the intestinal tract. However, it has been reported that certain members of enterococci can sporadically cause diarrhea in neonatal animals including piglets [24-27]. The pathogenic potential of enterococci seems to be associated with their ability to intimately adhere to the intestinal epithelium but the mechanisms by which these bacteria cause diarrhea remain unclear. So far, no evident mucosal damage has been reported in association with enterococci infection. In this study we also observed adhesion of *Enterococcus* spp. to the intestinal epithelial cells in the diarrheic piglets, which suggests pathogenic ability of these bacteria. A significant positive correlation between the presence of enterococci and histological lesions (villous atrophy and mild epithelial lesions) can be explained by the fact that the positive fluorescence signals for adherent *Enterococcus* spp. were seen in the small intestine of piglets that also had adherent *E. coli.* If these lesions were a consequence of a bacterial infection, they should be associated with *E. coli* rather than *Enterococcus* spp. as discussed above. Nevertheless, simultaneous colonization of the intestinal mucosa surface by these bacteria is an interesting finding and suggests their close interactions. Previously, a virulent synergistic effect between *E. coli* and *E. faecalis* has been described in relation to experimental polymicrobial infections [28] and it has been suggested that *E. faecalis* may inhibit phagocytosis of other pathogens including *E. coli*, and prevent them from intracellular death [29].

The majority of the piglets positive for adherent *E. coli* and adherent enterococci belonged to the same herd, which indicates that environmental factors influence composition of intestinal microbiota and eventual pathogens. This finding emphasizes the complexity of pathogenesis of porcine neonatal diarrhea and suggests that consideration of herd related aspects may be crucial for diagnosis and control of diarrheic conditions in piglets.

*C. perfringens* type A and *C. difficile* are nowadays regarded as ones of the most common bacterial species involved in pig neonatal diarrhea worldwide [30]. In this study, the occurrence of *C. perfringens* and its amount detected by FISH were similar in diarrheic and non-diarrheic piglets. Pathologically, degenerative and necrotic changes in the intestinal mucosa are commonly associated with clostridial enteritis and the bacteria are usually present among the necrotic tissue [30]. Such lesions were observed only in one diarrheic piglet in this study and it has been confirmed by microbiological testing that this piglet was positive for *C. perfringens* type C [3], which in that case can be regarded as a cause of enteritis. However, the mechanisms that could be involved in *C. perfringens* type A infection, remain unclear and there is no certain evidence for an adhesion of this bacterium to not destroyed intestinal tissue. Only few studies have investigated *C. perfringens* type A adhesive properties [31,32], but their results were inconclusive and to date, it is generally believed that *C. perfringens* does not have the ability to adhere to healthy intestinal epithelium. In agreement with this, the presence of *C. perfringens* in close proximity to the mucosal surface was seen in similar prevalence in both groups of piglets in this study (20 % diarrheic vs. 30 % non-diarrheic) and did not correlate with histological lesions, suggesting that the localization of *C. perfringens* cells in the intestinal mucosa is not linked to its pathogenicity. However, the pathogenesis of clostridial enteritis is commonly associated with the ability to produce toxins and diagnosis of the infection is based on the detection of large numbers of toxigenic bacteria [30]. Therefore, the role of *C. perfringens* type A should not be definitely ruled out and the determination of the importance of this bacterium in neonatal diarrhea should be supported by thorough investigation on clostridial toxins.

*C. difficile* infections are currently reported as one of the most common causes of pig neonatal diarrhea in some countries and whenever diagnosed, the culturing reveals heavy growth of this bacterium [33]. Microscopically, *C. difficile* infection is characterized by catarrhal, fibrinous or purulent colitis, however such lesions were not observed in this study. Furthermore, there was no association between the presence of this bacteria and pathological changes in the colon. Moreover, the occurrence of *C. difficile* and its amount did not differ significantly between diarrheic and non-diarrheic piglets. Therefore the presence of this bacterium seems not to be linked to the investigated diarrhea and these results are in agreement with other reports [34]. Additional studies with focus on clostridial toxins are being conducted to determine the role of both *Clostridia* species in the pathogenesis of presently reported neonatal diarrhea.

## Conclusions

Based on the results of this study, we conclude that potentially pathogenic bacteria such as non-ETEC *E. coli* and *Enterococcus* spp. might be involved in neonatal diarrhea in pigs less than 1 week old. Further identification of these bacteria is necessary in order to determine their pathogenicity and role in this syndrome. These bacteria should be taken into consideration whenever diagnosis of the pathogens commonly associated with neonatal porcine diarrhea fails.

### Animal ethics

The study was conducted in compliance with general ethical principles and with informed client consent. All farms providing animals presented high standards of veterinary care.

## Competing interests

The authors of this research paper have no competing interests.

## Authors’ contributions

BJ, MB and TKJ participated in the conception and design of this study and contributed to the interpretation of the results. Histopathological and FISH investigation and drafting of the manuscript were performed by BJ. MB and TKJ revised the manuscript for intellectual content. AS performed statistical analyses. All authors participated in proofreading and approval of the final manuscript.
